# The efficacy and safety of ATR inhibitors in the treatment of solid tumors: a systematic review and meta-analysis

**DOI:** 10.3389/fonc.2025.1706837

**Published:** 2025-12-02

**Authors:** Yaxu Su, Xinyu Lu, Zhenzhen Bu, Xue Yang, Ping Liu

**Affiliations:** 1 Department of Obstetrics and Gynecology, Northern Jiangsu People’s Hospital, Yangzhou, Jiangsu, China; 2 Guangxi Medical University, Nanning, Guangxi, China; 3 Department of Oncology, Northern Jiangsu People’s Hospital, Yangzhou, Jiangsu, China; 4 Department of Radiation, The Affiliated Hospital of Xuzhou Medical University, Xuzhou, Jiangsu, China

**Keywords:** ATR inhibitor, neoplasms, tumor, meta-analysis, progression-free survival, adverse events

## Abstract

Ataxia telangiectasia and Rad3-related kinase (ATR) is a central regulator of the DNA damage response and a promising therapeutic target in tumors with genomic instability. This meta-analysis evaluated the efficacy and safety of ATR inhibitors in patients with solid tumors. A systematic search of PubMed, Embase, and major Chinese and international databases up to September 2025 identified ten clinical trials including 810 patients treated with ATR inhibitors as monotherapy or in combination. Outcomes assessed included objective response rate, disease control rate, progression-free survival, overall survival, and treatment-related adverse events. Pooled analyses showed no significant overall improvement in efficacy endpoints compared with standard therapies. However, subgroup analysis demonstrated marked benefit in non-small cell lung cancer, with significantly prolonged progression-free survival and overall survival, while efficacy in other tumor types was minimal. The overall risk of adverse events was comparable to control groups, although hematologic toxicities such as myelosuppression (pooled RR = 1.04) and neutropenia (pooled RR = 1.34) were common. These findings suggest that ATR inhibitors may be particularly effective in non-small cell lung cancer and selected ovarian cancer subgroups, but further biomarker-driven randomized trials are required to confirm their clinical value and optimize patient selection.

**Systematic Review Registration:**

https://www.crd.york.ac.uk/prospero/, identifier CRD420251123612.

## 1 Introduction

The DNA damage response (DDR) is a signaling network that preserves genomic stability by repairing DNA lesions, regulating the cell cycle, and inducing apoptosis, thereby preventing mutagenesis and tumorigenesis (1, 2). The principal regulators of DDR are members of the phosphatidylinositol 3-kinase-related kinase (PIKK) family, notably ataxia-telangiectasia mutated (ATM) and ATM- and Rad3-related (ATR) (3). As a pivotal DDR kinase, ATR orchestrates cell-cycle checkpoints and coordinates DNA repair by activating multiple downstream pathways. Dysregulated ATR expression can promote tumor progression and confer resistance to diverse anticancer therapies. This aberrant signaling has been linked to the initiation, development, and poor prognosis of malignancies including ovarian cancer, breast cancer, and melanoma (4–8).

Given its central role, ATR has become a compelling therapeutic target, especially in tumors with ATM deficiency or homologous recombination repair (HRR) defects (9). ATR inhibitors selectively suppress tumor growth by potently blocking ATR kinase activity, thereby disrupting DDR and impairing cell-cycle regulation (10, 11). Preclinical and early clinical studies have shown synergistic activity when ATR inhibitors are combined with chemotherapy, radiotherapy, immunotherapy, or poly(ADP-ribose) polymerase (PARP) inhibitor (4).

Several ATR inhibitors are in clinical development, including Berzosertib (M6620), Ceralasertib (AZD6738), Elimusertib (BAY-1895344), and Camonsertib (RP-3500) (12, 13). Berzosertib, the first ATR inhibitor to enter human trials, blocks the ATR–CHK1 signaling axis and enhances DNA damage. The pivotal NCT02487095 trial (14) demonstrated that Berzosertib combined with chemotherapy improved short-term efficacy in platinum-resistant small cell lung cancer, with manageable toxicity. Subsequent studies further evaluated its safety, efficacy, and radiosensitizing potential (15, 16). Parallel trials with other ATR inhibitors, including Ceralasertib, Elimusertib, Gartisertib, and M1774, have reported favorable tolerability and encouraging antitumor activity in triple-negative breast cancer, ovarian cancer, and non-small cell lung cancer (17–19).

Despite these advances, challenges remain. ATR is essential for cell viability, and its inhibition can trigger severe hematologic toxicity such as myelosuppression. Early-generation inhibitors (e.g., NU6027 and AZ20) were abandoned due to poor selectivity, whereas newer agents like Berzosertib and Ceralasertib, though more specific, suffer from suboptimal solubility and metabolite accumulation (12, 20). Moreover, the lack of robust predictive biomarkers hampers patient stratification and limits precision in clinical applications.

To provide a comprehensive evaluation of ATR inhibitors in solid tumors, we performed a systematic review and meta-analysis, synthesizing clinical trial data on key efficacy outcomes—objective response rate (ORR), disease control rate (DCR), progression-free survival (PFS), overall survival (OS)—and treatment-related adverse events (AEs). The aim was to define the therapeutic potential and limitations of ATR inhibitors, thereby informing clinical decision-making and guiding future research.

## 2 Methods

This meta-analysis followed the Preferred Reporting Items for Systematic Reviews and Meta-Analyses (PRISMA) guidelines, and the protocol was registered in PROSPERO (registration ID: CRD420251123612). The registration record is publicly accessible at: https://www.crd.york.ac.uk/prospero/display_record.php?ID=CRD420251123612. The study protocol was registered in PROSPERO on August 10, 2025—this is a pre-hoc registration.

### 2.1 Search strategy

We conducted a comprehensive search for clinical trials evaluating ATR inhibitors, including Berzosertib (M6620), Ceralasertib (AZD6738), Elimusertib (BAY-1895344), Camonsertib (RP-3500), and related compounds. Databases searched were PubMed, Web of Science, Cochrane Library, Embase, Chinese National Knowledge Infrastructure (CNKI), Wanfang Data, Chinese Biological Medicine Database, and VIP Database, with a cutoff of September 2025. Abstracts from major oncology meetings—including the American Society of Clinical Oncology (ASCO), the American Association for Cancer Research (AACR), and the European Society for Medical Oncology (ESMO)—were also screened. All retrieved records were aggregated and imported into NoteExpress. Duplicates were first removed using the software’s automated tool, followed by a manual screening of titles and abstracts by two independent reviewers to identify and discard any remaining duplicates. No language restrictions were applied during the literature search or study selection process.

### 2.2 Eligibility criteria

Studies were included if they (1) were clinical trials of ATR inhibitors as monotherapy or combination therapy; (2) reported clinical outcomes such as ORR, DCR, PFS, OS, or AEs; (3) used randomized controlled trial (RCT), quasi-RCT, or non-randomized comparative designs (NRCT), where “NRCT” were defined as comparative studies where intervention and control groups were non-randomly allocated but were systematically matched or statistically adjusted for key baseline characteristics; and (4) enrolled at least 10 patients. Exclusion criteria: Studies were excluded if they (1) were duplicate publicatins; (2) had incomplete or inconsistent data that could not support analysis; (3) were inconsistent with the required literature type (including reviews, basic research such as animal experiments and *in vitro* studies, case reports, commentaries, letters to the editor, or clinical guidelines); (4) had unqualified study designs (including single-arm studies without concurrent placebo or positive control groups, and non-comparative studies not designed to compare intervention effectiveness); (5) were only available as conference abstracts or clinical trial registration information with no accessible complete data (after attempting to contact corresponding authors for full texts or raw data); or (6) failed to report pre-defined key outcome measures (ORR, DCR, PFS, OS, or AEs) or lacked usable data for Meta-analysis.Two reviewers independently screened and extracted data. Ultimately, 10 trials comprising 810 patients were included.

### 2.3 Data extraction and quality assessment

Information collected included study design, demographics, tumor type, intervention regimen, and clinical outcomes. RCTs were assessed using the Cochrane risk-of-bias tool, and non-RCTs were evaluated with the Newcastle–Ottawa Scale (NOS). The quality rating of outcome measures were based on the GRADE framework. All studies demonstrated adequate methodological rigor.

### 2.4 Statistical analysis

Meta-analyses were performed with RevMan 5.4 and R 4.5.1. Effect sizes were calculated as risk ratios (RRs) or hazard ratios (HRs) with 95% confidence intervals (CIs). Heterogeneity was assessed using Cochran’s Q test and the I² statistic. Publication bias was evaluated with Egger’s test for continuous outcomes and Begg’s test for dichotomous outcomes. Sensitivity analyses were conducted to test result robustness. A p-value <0.05 was considered statistically significant.

## 3 Results

### 3.1 Overview of included studies

**Figure 1:**
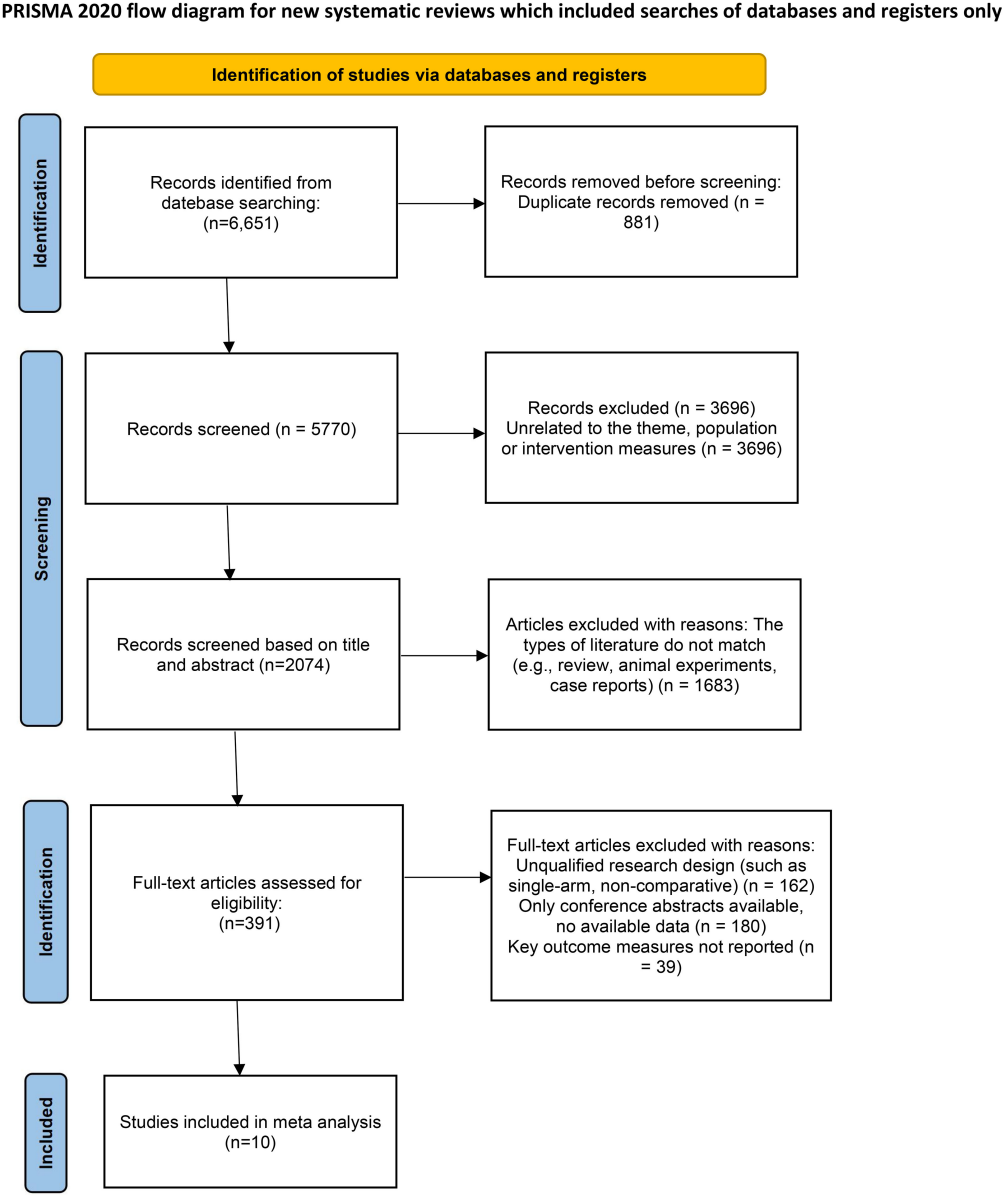
Flow chart of the meta-analysis.

A total of 6, 651 records were retrieved from eight databases (PubMed, Embase, Web of Science, SinoMed, Wanfang Data, VIP Database, Chinese National Knowledge Infrastructure [CNKI], and the Cochrane Library) up to September 2025. After removing 881 duplicates, 3, 696 irrelevant records, and 1, 683 reviews, animal studies, or case reports, 391 full-text articles were assessed. Of these, 381 were excluded due to inconsistent outcomes (n=39), methodological limitations (n=162), or unpublished data (n=180). Ultimately, 10 clinical trials involving 810 patients were included (**Figure 1**).

**Figure 2:**
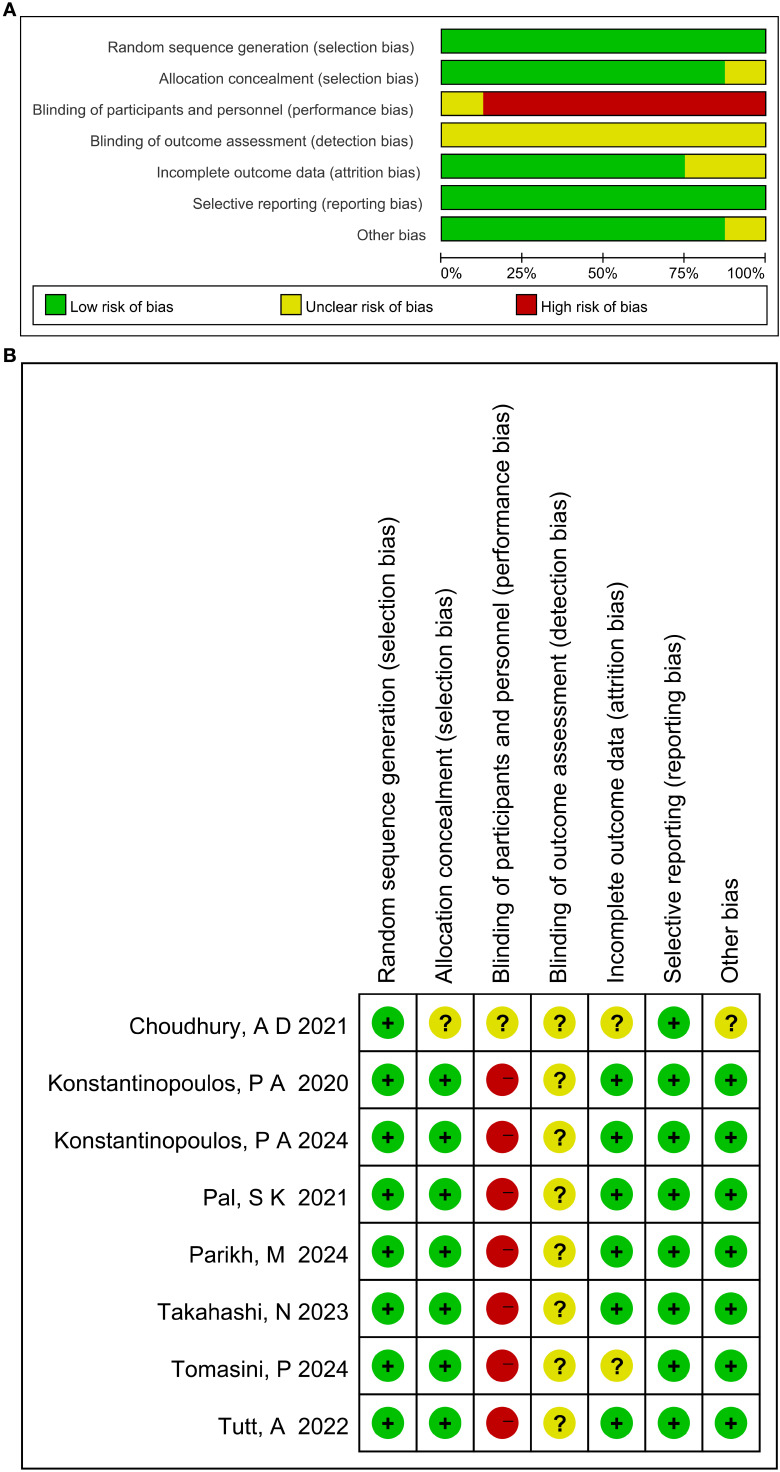
Assessment of risk of bias in the included studies (**A**: Summary across studies; **B**: Individual study profiles). Colors denote risk: green=low, yellow=unclear, red=high.

Among the included studies, eight were randomized controlled trials (RCTs) and two were non-randomized controlled trials (non-RCTs). Following quality assessment, all studies demonstrated generally high methodological quality. The evaluation results for RCTs are shown in **Figure 2**, while those for non-RCTs are presented in the NOS column of **Table 1**. The RoB 2 domain-level judgments for RCTs, specific NOS scores for non-RCTs, and summary of findings (SoF) table are shown in **Supplementary Table S1**. Most were Phase II trials, with one classified as a Phase Ib/IIa trial. The primary tumor types investigated included non-small cell lung cancer (NSCLC), ovarian cancer, and urothelial carcinoma. A summary of trial characteristics is provided in **Table 2**.

**Table 1:** Interventions and methodological quality (Newcastle–Ottawa Scale) of non-randomized studies.

First author, year	Intervention		NOS	(Refs.)
	Experimental	Control		
Konstantinopoulos, P A 2024	gemcitabine + berzosertib	gemcitabine	*	(42)
Parikh, M 2024	cisplatin 60 mg/m² iv d1 + gemcitabine 875 mg/m² iv d1/8 + berzosertib 90 mg/m² iv d2/9, 21-day cycle	cisplatin 70 mg/m² iv d1 + gemcitabine 1000 mg/m² iv d1/8, 21-day cycle	*	(43)
Park, Sehhoon 2024	olaparib 300 mg po BID + ceralasertib 160 mg po QD d1-7, 28-day cycle	olaparib 300 mg po BID, 21-day cycle	7	(44)
Tomasini, P 2024	Durvalumab + Ceralasertib	Docetaxel	*	(45)
Takahashi, N 2023	topotecan 1.25 mg/m² ivgtt d1-5 + berzosertib 210 mg/m² ivgtt d2/5, 21-day cycle	topotecan 1.25 mg/m² ivgtt d1-5, 21-day cycle	*	(46)
Tutt, A 2022	olaparib 300 mg BID + ceralasertib 160 mg d1-7, 28-day cycle	olaparib 300 mg BID, 28-day cycle	*	(47)
Choudhury, A D 2021	berzosertib 90 mg/m² d 2/9 + carboplatin (carbo) AUC 5 d1	docetaxel 60 mg/m² Day 1 + carboplatin (carbo) AUC 4 d1	*	(48)
Pal, S K 2021	cisplatin 60 mg/m² d1 + gemcitabine 875 mg/m² d1/8 + berzosertib 90 mg/m² d2/9, 21-day cycle	cisplatin 70 mg/m² d1 + gemcitabine 1000 mg/m² d1/8, 21-day cycle	*	(49)
Park, S 2021	olaparib + ceralasertib	olaparib	7	(50)
Konstantinopoulos, P A 2020	gemcitabine 1000 mg/m² iv d1/8 + berzosertib 210 mg/m² iv d2/9, 21-day cycle	gemcitabine 1000 mg/m² iv, 21-day cycle	*	(51)

**Table 2:** Characteristics of randomized and non-randomized controlled clinical trials included in the meta-analysis.

First author, year	Phase	Design	Type of inhibitors	Type of tumors	Total samples	Age, years (range)	Male sex, n(%)	(Refs.)
Konstantinopoulos, P A 2024	II	RCT	Berzosertib(M6620)	Ovarian cancer	34/36	NA	NA	(42)
Parikh, M 2024	II	RCT	Berzosertib(M6620)	Urothelial Carcinoma	46/41	67	NA	(43)
Park, Sehhoon 2024	II	NRCT	Ceralasertib(AZD6738)	Small cell lung cancer	26/15	66 (47–78)/68(48-76)	24(92.3)/14(93.3)	(44)
Tomasini, P 2024	Ib/IIa	RCT	Ceralasertib(AZD6738)	Non-small cell lung cancer	32/31	NA	NA	(45)
Takahashi, N 2023	II	RCT	Berzosertib(M6620)	Small cell lung cancer	40/20	60(46-79)/59(34-72)	24(60)/9(45)	(46)
Tutt, A 2022	II	RCT	Ceralasertib(AZD6738)	Triple-negative breast cancer	112/114	53	NA	(47)
Choudhury, A D 2021	II	RCT	Berzosertib(M6620)	prostate cancer	31/34	NA	NA	(48)
Pal, S K 2021	II	RCT	Berzosertib(M6620)	Urothelial Carcinoma	46/41	67.5(32-82)/65(32-84)	38(83)/8(17)	(49)
Park, S 2021	II	NRCT	Ceralasertib(AZD6738)	Small cell lung cancer	26/15	NA	NA	(50)
Konstantinopoulos, P A 2020	II	RCT	Berzosertib(M6620)	Ovarian cancer	34/36	NA	NA	(51)

### 3.2 Objective response rate

**Figure 3:**
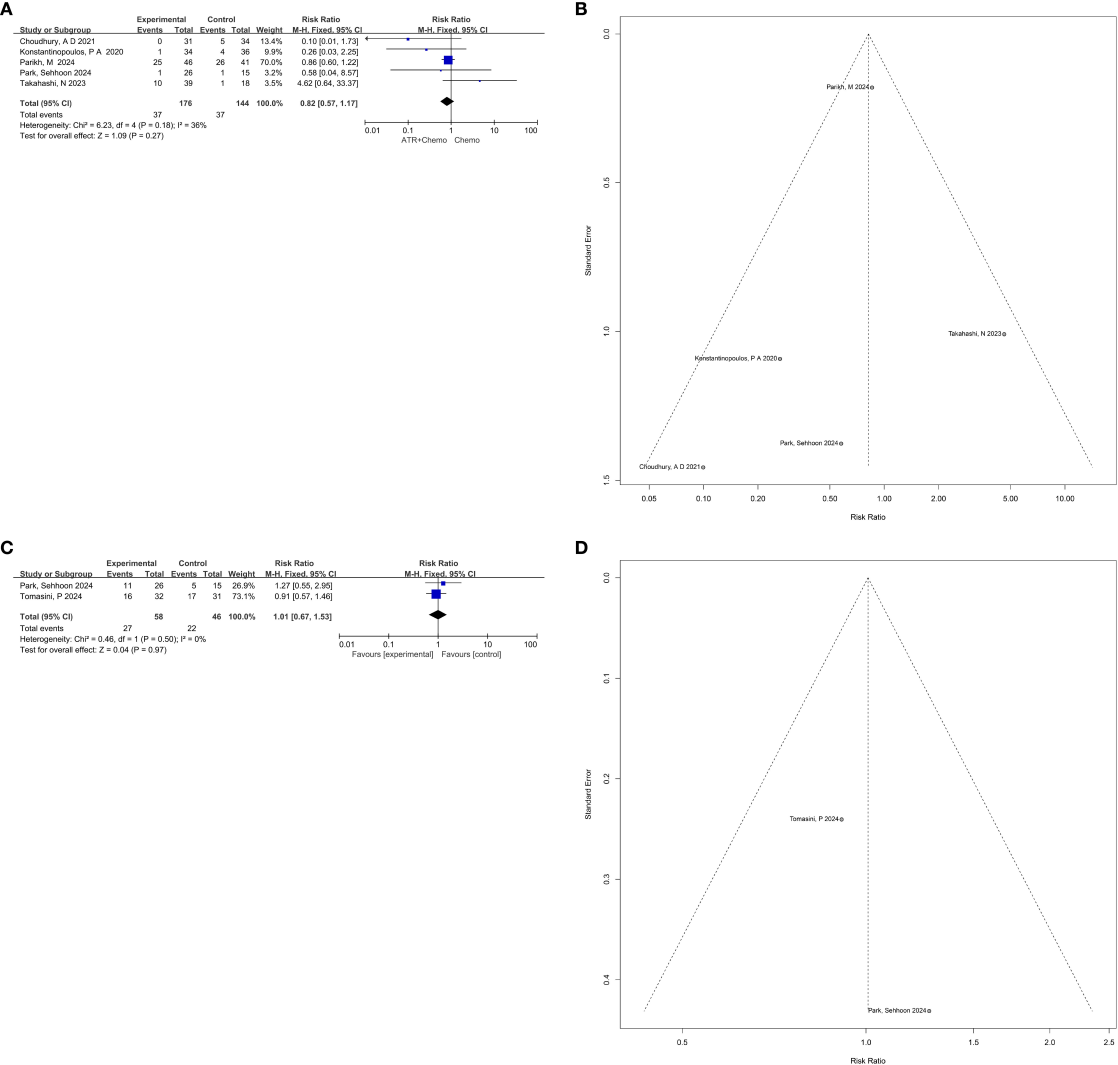
**(A)** Forest plot and **(B)** funnel plot depicting the pooled results for the overall response rate. **(C)** Forest plot and **(D)** funnel plot illustrating the pooled results for the disease control rate.

Five studies reported objective response rate (ORR). No significant heterogeneity was observed (p = 0.18, I² = 36%), so a fixed-effects model was applied. Pooled analysis showed no significant improvement in ORR between ATR inhibitor regimens and control arms (RR = 0.82, 95% CI: 0.57–1.17, p = 0.27) (**Figure 3A**). Funnel plot inspection revealed no asymmetry, further supported by Begg’s test (**Figure 3B**; **Table 3**), suggesting no statistically detectable publication bias. However, interpretation should remain cautious given the limited number of studies.

**Table 3:** Assessment of publication bias using Begg’s test.

Study identifier	Correlation_coefficient	Z_score	P_value
ORR	0.6	1.5	0.1336
DCR	1	47453132.81	0

Correlation Coefficient: Represents the Spearman’s rank correlation coefficient estimated by Begg’s test, quantifying the strength and direction of the association between the ranks of absolute effect sizes and the ranks of their standard errors. A non-zero correlation suggests potential publication bias (e.g., smaller studies with more extreme effect sizes).

Z-score: The z-statistic calculated from the Spearman’s correlation coefficient, used to test whether the observed correlation differs significantly from zero. It follows a standard normal distribution under the null hypothesis (no publication bias).

P-value: The p-value associated with the z-statistic, assessing the statistical significance of the observed correlation. A p-value < 0.05 is typically interpreted as evidence of potential publication bias, while a p-value ≥ 0.05 indicates no sufficient evidence for publication bias.

### 3.3 Disease control rate

Two studies reported disease control rate (DCR). Pooled analysis indicated virtually no difference between ATR inhibitor regimens and control groups (RR = 1.01, 95% CI: 0.67–1.53, p = 0.97), with no heterogeneity (I² = 0%, p = 0.50) (**Figure 3C**). Funnel plot and Begg’s test suggested potential publication bias, but the trim-and-fill method could not be reliably applied due to the small number of included studies (**Figure 3D**). Crucially, statistical testing for publication bias is unreliable with only two studies; therefore, the risk of publication bias for DCR cannot be reliably assessed or excluded.

### 3.4 Progression-free survival

**Figure 4:**
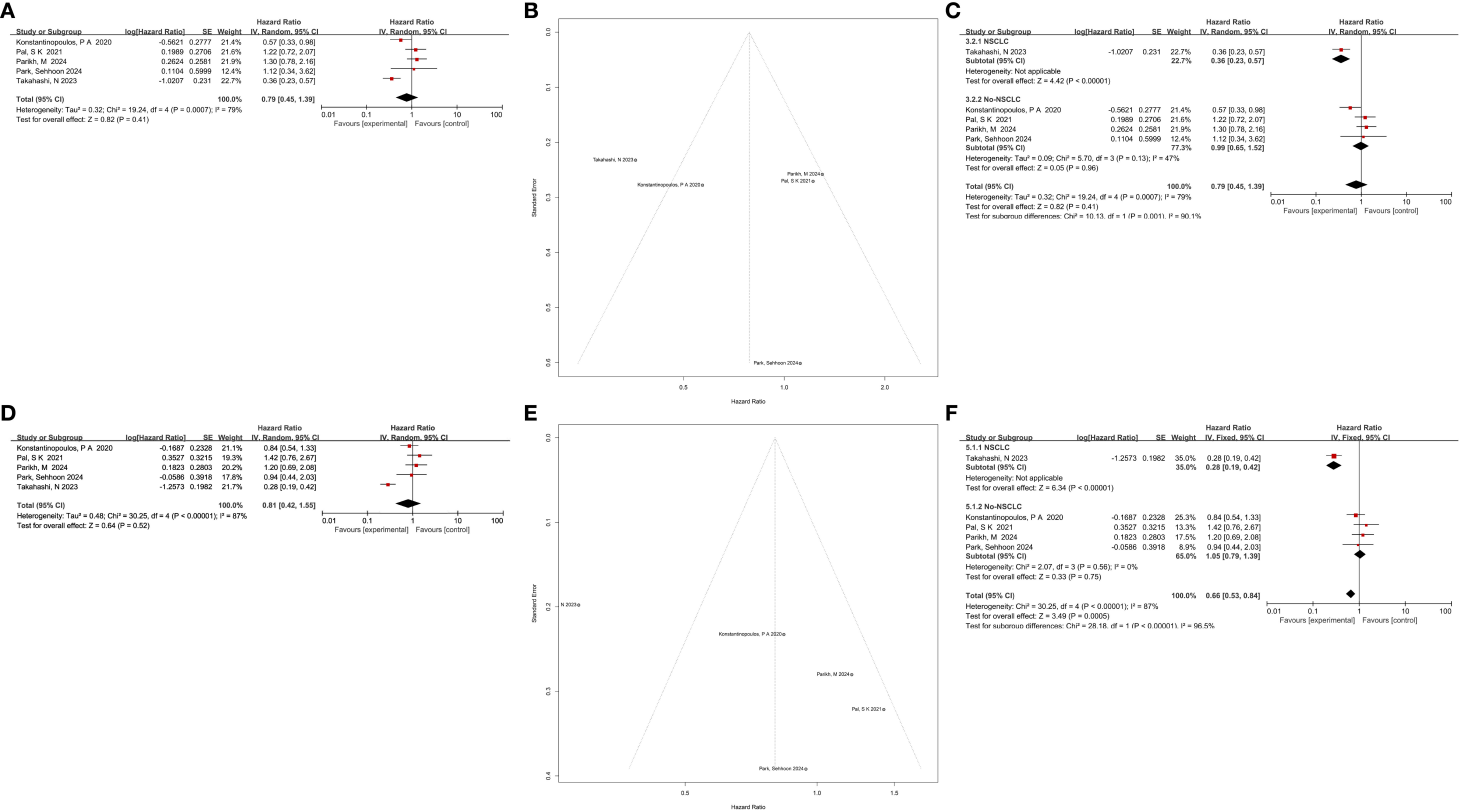
**(A)** Forest plot and **(B)** funnel plot illustrating the pooled results for progression-free survival (PFS). **(C)** Subgroup analysis of PFS in patients with and without non-small cell lung cancer (NSCLC). **(D)** Forest plot and **(E)** funnel plot illustrating the pooled results for overall survival (OS). **(F)** Subgroup analysis of PFS in patients with and without NSCLC.

Eight trials reported progression-free survival (PFS), but only five provided sufficient hazard ratio (HR) data for meta-analysis. The pooled HR was 0.79 (95% CI: 0.45–1.39, p = 0.41) (**Figure 4A**). While the experimental arms suggested a trend toward improved PFS, substantial heterogeneity was detected (p = 0.0007, I² = 79%). Funnel plot and Egger’s test indicated no publication bias (**Figure 4B**; **Table 4**). Nevertheless, given the small number of trials, the power of these tests is limited, and the absence of bias cannot be definitively excluded.

**Table 4:** Results of Egger’s test for publication bias.

Study identifier	Slope (coefficient)	Standard error	t-score	P-value
mOS	5.3538	3.6331	1.4736	0.237
mPFS	1.3391	1.9752	0.6779	0.5464

Slope (Coefficient): Represents the coefficient of the regression line estimated by Egger’s test, indicating the degree of correlation between effect sizes and their standard errors.

Standard Error: The standard error of the regression slope, indicating the precision of the estimated coefficient.

t-Score: The t-statistic associated with the regression coefficient, used to determine if the slope significantly differs from zero (indicating potential publication bias).

P-Value: The p-value associated with the t-statistic, which assesses the statistical significance of the findings. A p-value below a certain threshold (commonly 0.05) indicates significant publication bias.

Subgroup analysis demonstrated that NSCLC patients derived significant benefit (HR = 0.36, 95% CI: 0.23–0.57, p < 0.00001), whereas non-NSCLC patients did not (HR = 0.99, 95% CI: 0.65–1.52, p = 0.96) (**Figure 4C**). Sensitivity analysis excluding Takahashi et al. (2023) reduced heterogeneity from 79% to 47%, suggesting that this study was the primary driver of variability.

### 3.5 Overall survival

Five studies reported overall survival (OS). The pooled HR was 0.81 (95% CI: 0.42–1.55, p = 0.52) (**Figure 4D**), indicating no significant difference between experimental and control groups. No publication bias was detected by Begg’s or Egger’s tests (**Figure 4E**). Similar to PFS, the limited number of studies means these results must be interpreted with extreme caution due to low statistical power.

Subgroup analysis revealed pronounced benefit in NSCLC (HR = 0.28, 95% CI: 0.19–0.42, p < 0.00001), whereas non-NSCLC subgroups showed no improvement (HR = 1.03, 95% CI: 0.69–1.54, p = 0.91) (**Figure 4F**). Sensitivity analysis demonstrated that exclusion of Takahashi et al. (2023) reduced heterogeneity from 87% to 0%, confirming its influence on overall results.

### 3.6 Safety and adverse events

**Figure 5:**
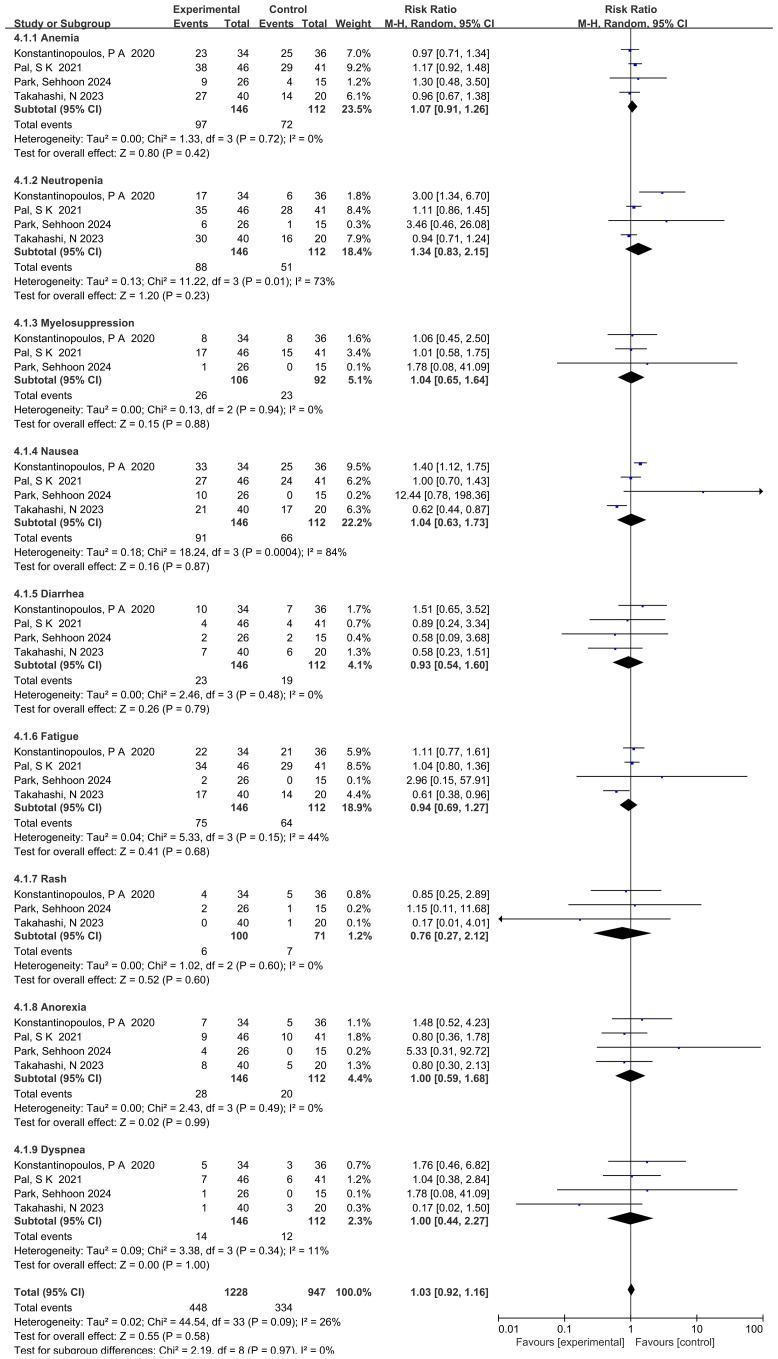
Forest plot showing the pooled results of adverse events (AEs).

All included trials reported adverse events (AEs). Common treatment-related AEs included anemia, neutropenia, myelosuppression, nausea, diarrhea, fatigue, rash, anorexia, and dyspnea. Pooled analysis showed no overall increase in AE risk with ATR inhibitors compared to controls (RR = 1.03, 95% CI: 0.92–1.16, p = 0.58) (**Figure 5**).

Notably, neutropenia (pooled RR = 1.34, 95% CI: 0.83–2.15, p = 0.23, I² = 73%) and nausea (pooled RR = 1.04, 95% CI: 0.63–1.73, p = 0.87, I² = 84%) demonstrated high heterogeneity. This variability may reflect differences in drug dosages, baseline immune function, subjective toxicity assessments, and follow-up durations across studies. Despite these variations, no statistically significant differences were observed.

### 3.7 Sensitivity analysis

Sequential exclusion of individual studies showed stable results for most outcomes, confirming the robustness of pooled estimates. The main exceptions were PFS and OS, where heterogeneity was notably influenced by Takahashi, N 2023 (**Supplementary Figure S1**).

## 4 Discussion

### 4.1 Summary of main findings

This meta-analysis, which included 8 randomized controlled trials and 2 non-randomized comparative studies involving 810 patients, showed that when all tumor types were pooled, ATR inhibitors did not significantly improve key efficacy outcomes compared with conventional therapies. However, stratified analysis revealed that ATR inhibitors achieved favorable objective response rates (ORR) and significantly reduced hazard ratios (HRs) for median progression-free survival (PFS) and overall survival (OS) in non-small cell lung cancer (NSCLC). By contrast, in tumor types other than NSCLC, the efficacy was disappointing and even associated with a potential increase in adverse events. These findings suggest that the therapeutic potential of ATR inhibition may be restricted to specific tumor subtypes.

### 4.2 Biological rationale and interpretation

The observed pattern—marked efficacy in NSCLC but limited activity in other cancers—is biologically plausible and hinges on the principle of “ATR dependency”.

In NSCLC, this vulnerability is driven by specific co-mutations. KRAS mutations drive oncogenic replicative stress, while concurrent loss of STK11/LKB1 and/or KEAP1 (21)creates metabolic dysregulation and further oxidative stress. To survive this profound, high-stress state, these cancer cells become compensatorily and critically dependent on the ATR–CHK1 signaling pathway for DNA repair and genomic stability (22, 23). ATR inhibitors, such as ceralasertib, block this critical compensatory mechanism, resulting in replication fork collapse and mitotic catastrophe (24). Clinically, patients with NSCLC harboring these co-mutations (KLK-subtype) treated with ceralasertib combinations achieved a median PFS of 8.4 months and an ORR of 35%, strongly supporting this mechanistic model (25).

This principle of “ATR dependency” is not unique to NSCLC; it also explains the outcomes observed in specific subgroups of ovarian cancer. In high-grade serous ovarian cancer, this vulnerability is often driven by defects in homologous recombination (HR), such as BRCA1/2 mutations, or BRCA reversion mutations that confer PARPi resistance (26–28). These HR-deficient states create a synthetic lethal reliance on ATR for survival. Furthermore, another well-defined subgroup exhibits CCNE1 amplification, which induces severe, unscheduled replicative stress, forcing a similar dependency on ATR signaling to prevent mitotic catastrophe (29, 30).

In stark contrast, the poor efficacy in most other solid tumors likely reflects the absence of a pre-existing, critical ATR dependency. These tumors often possess a robust and redundant network of DDR pathways (e.g. functional ATM and HR pathways) (9). Furthermore, their baseline level of replication stress may be insufficient to induce cellular collapse when the ATR pathway alone is inhibited. The clinical benefit rate of ATR inhibitor monotherapy in some ATM wild-type tumors, for instance, is below 10%, further supporting this mechanistic explanation (18, 31).

Beyond its canonical role in DDR and genomic stability, ATR also participates in multiple non-nuclear cellular processes that contribute to tumor survival, such as regulating mitochondrial bioenergetics, maintaining cytoskeletal integrity, and modulating metabolic flux (32–34). These non-DDR functions are increasingly recognized as potential therapeutic targets, but current clinical ATR inhibitors (e.g., ceralasertib, berzosertib) are specifically designed to block ATR’s kinase activity in the DDR pathway, with minimal impact on its other cellular roles. This specificity explains why these agents fail to exert efficacy in most solid tumors—without pre-existing DDR dependency, the untargeted non-DDR functions of ATR cannot be exploited to induce tumor cell death, and redundant DDR networks further compensate for ATR inhibition.

### 4.3 Comparison with prior evidence and role relative to PARP inhibitors

Our findings align with and contextualize the results from early-phase, single-arm trials. For example, Shah et al. and Konstantinopoulos et al. reported promising DCRs of 50–75% in ovarian cancer (35, 36). Our analysis of comparative trials helps quantify this benefit, showing a modest ORR of ~22% in BRCA-mutant ovarian cancer, which is comparable to PARP monotherapy (37, 38).

A critical question for the clinical development of ATR inhibitors is their relationship with PARP inhibitors (PARPi), particularly in BRCA-mutant cancers, where PARPi are an established standard of care (39). Our systematic review did not identify any head-to-head trials comparing ATRi directly to PARPi, precluding any conclusions on comparative efficacy. However, their mechanisms are distinct. PARPi trap PARP onto DNA, which is particularly toxic in the context of HRD (synthetic lethality) (40). In contrast, ATRi block the activation of the G2/M checkpoint, forcing cells with high replicative stress into premature mitotic catastrophe (41). This mechanistic distinction suggests potential for ATRi in PARPi-resistant settings. Critically, the report that 18% of patients with BRCA reversion mutations (a common PARP resistance mechanism) achieved durable disease control with ATR inhibitor combination therapy (37) reinforces this very principle: ATR inhibitors offer a viable therapeutic option for PARP-resistant populations.

### 4.4 Predictive biomarkers and mechanisms of resistance

The success in NSCLC and ovarian cancer subgroups underscores that ATR inhibitors must be developed as a biomarker-driven therapy. Our finding in NSCLC is promising but broad; a more precise predictive biomarker is needed. Potential markers include direct evidence of pathway activation, such as high baseline levels of pATR or its downstream target pCHK1, which may indicate an “ATR-addicted” state.

Beyond pathway activity, synthetic lethality markers remain the most promising. As discussed, defects in the HR pathway (BRCA1/2) are a key predictor. Similarly, loss of ATM function (common in many cancers) forces a compensatory reliance on the ATR pathway, creating another potent synthetic lethal context. Understanding mechanisms of acquired resistance is also crucial, as preclinical models suggest bypass signaling via parallel checkpoint pathways, such as WEE1, as a potential escape mechanism.

### 4.5 Clinical and translational implications

The heterogeneity of clinical outcomes across tumor types indicates that ATR inhibitors are unlikely to serve as broadly effective anticancer agents. Instead, their value lies in treating a subset of refractory tumors with distinct genomic alterations. Translationally, these findings emphasize the importance of biomarker-driven patient selection and support integrating ATR inhibitors into the precision oncology paradigm.

### 4.6 Strengths and limitations

The strengths of this meta-analysis include the high quality of included trials, rigorous risk-of-bias assessment, the clinical relevance and updated recent trial data, and comprehensive evaluation of multiple endpoints. Importantly, the lack of benefit in non-NSCLC tumors reinforces the conclusion that ATR inhibitors cannot be considered as broad-spectrum anticancer agents.

Nonetheless, several limitations must be acknowledged. First, long-term survival data are scarce, with few trials reporting outcomes beyond three years. Second, although subgroup analyses suggest meaningful benefit in NSCLC, the number of eligible trials was small, limiting the robustness of conclusions. Third, the majority of included studies were Phase I/II trials, which are prone to overestimating efficacy and early-phase bias due to selective patient enrollment and short follow-up periods. Meanwhile, the lack of consistent, prospective biomarker stratification across all tumor types hinders our ability to precisely define the most responsive patient population. Fourth, although formal statistical tests (Begg’s or Egger’s tests) for outcomes with N≥5 (ORR, PFS, OS) did not detect significant asymmetry, their power is substantially limited by the small number of included studies. Furthermore, for outcomes based on only two studies (e.g. DCR), publication bias cannot be excluded. The absence of mature overall survival (OS) data combined with biomarker inconsistency across studies collectively limit the clinical extrapolation of our findings to broader patient populations.

### 4.7 Future research directions

Future studies should prioritize late-phase randomized controlled trials focusing on NSCLC and ovarian cancer to identify the patient subgroups most likely to benefit. Head-to-head comparisons with PARP inhibitors and dual DDR-targeting strategies are essential to determine optimal sequencing and combination regimens. Long-term follow-up is required to assess the durability of survival benefits and late toxicities. Moreover, with the growing number of targeted therapies in oncology, health economic analyses will be crucial for assessing cost-effectiveness and guiding clinical adoption.

## 5 Conclusion

This meta-analysis suggests that ATR inhibitors show particularly favorable efficacy in patients with non-small cell lung cancer, while also demonstrating promising potential in the treatment of ovarian cancer. Although encouraging, the current evidence is limited by heterogeneity, possible publication bias, and the small body of available evidence, underscoring the need for well-designed clinical trials to validate these findings and to further explore combination strategies for optimizing therapeutic outcomes.

## Data Availability

Publicly available datasets were analyzed in this study. This data can be found here: https://pubmed.ncbi.nlm.nih.gov/.
