# Supplementary material for: The efficacy and safety of ATR inhibitors in the treatment of solid tumors: a systematic review and meta-analysis
Source: Front Oncol. 2025 Dec 2;15:1706837. doi: 10.3389/fonc.2025.1706837 (PMC12705417; doi:10.3389/fonc.2025.1706837)
Supplement: Supplementary file 1 [file DataSheet1.docx]

Supplementary Material

## Supplementary Figures

A


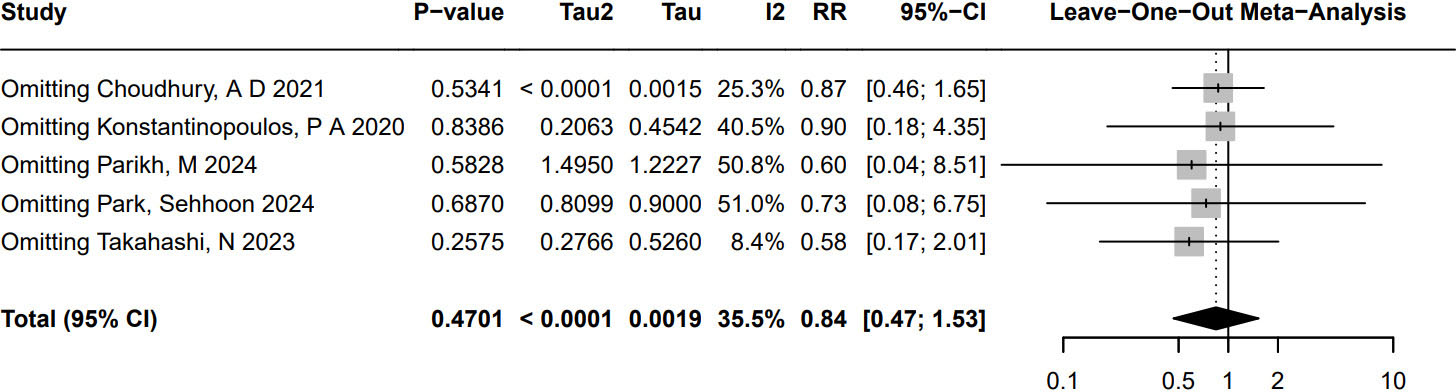


B


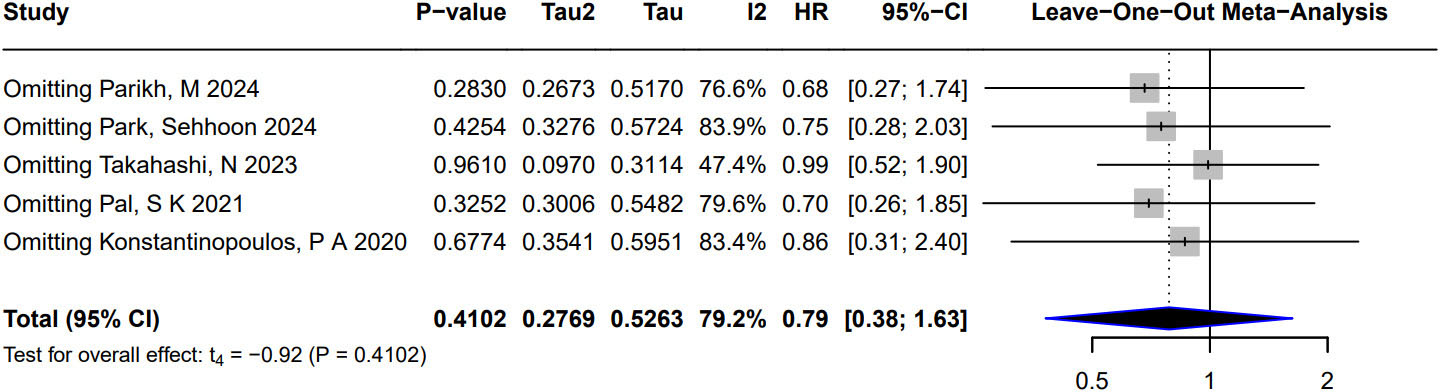


C


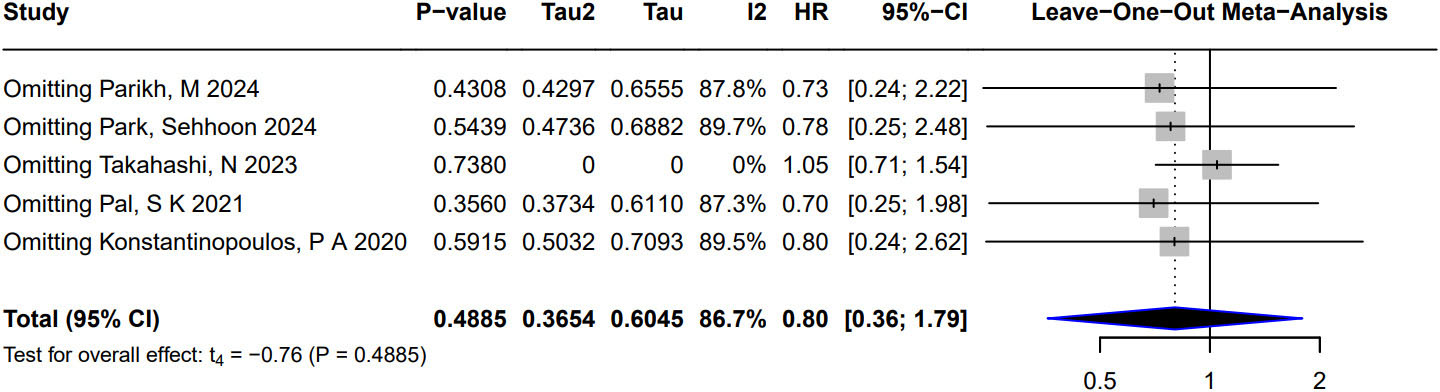


**Supplementary Figure 1.** Sensitivity analyses of ATR inhibitor efficacy across included studies. (A) Objective response rate (ORR). (B) Progression-free survival (PFS). (C) Overall survival (OS). Each panel shows the impact of sequential exclusion of individual studies on pooled effect estimates, indicating the robustness of the meta-analysis results.

## Supplementary Tables

**Supplementary Table 1**. (A) RoB 2 Domain-Level Judgments for RCTs. (B) NOS Item-Level Scores for Non-RCT). (C) summary of findings (SoF) for Outcomes.

A

| First author, year | D1: Randomization | D2: Deviation from intervention measures | D3: Missing outcome data | D4: Outcome measurement | D5: Selective Reporting | Overall risk |
| --- | --- | --- | --- | --- | --- | --- |
| Konstantinopoulos 2024 (21) | Low | Low | Low | Low | Low | Low |
| Parikh 2024 (22) | Low | Low | Low | Low | Low | Low |
| Tomasini 2024 (24) | Low | Low | Low | Low | Low | Low |
| Takahashi 2023 (25) | Low | Low | Low | Low | Some concerns | Some concerns |
| Tutt 2022 (26) | Low | Low | Low | Low | Low | Low |
| Choudhury 2021 (27) | Low | Low | Low | Low | Low | Low |
| Pal 2021 (28) | Low | Low | Low | Low | Low | Low |
| Konstantinopoulos 2020 (30) | Low | Low | Low | Low | Low | Low |

B

| First author, year | Selection | Comparability | Outcome | Total Score |
| --- | --- | --- | --- | --- |
| Park, S. 2024 (23) | 1. Representativeness of the exposed cohort : 1  2. Selection of the non exposed cohort: 1  3. Ascertainment of exposure: 1  4. Demonstration that outcome of interest was not present at start of study : 1 | 1. Comparability of cohorts on the basis of the design or analysis : 1 | 1. Assessment of outcome : 1 2. Was follow-up long enough for outcomes to occur: 1  3. Adequacy of follow up of cohorts:0 | 7/9 |
| Park, S. 2021 (29) | 1. Representativeness of the exposed cohort : 1  2. Selection of the non exposed cohort: 1  3. Ascertainment of exposure: 1  4. Demonstration that outcome of interest was not present at start of study : 1 | 1. Comparability of cohorts on the basis of the design or analysis : 1 | 1. Assessment of outcome : 1 2. Was follow-up long enough for outcomes to occur: 1  3. Adequacy of follow up of cohorts:0 | 7/9 |

C

| Outcomes | Control Group Risk | Relative Effect (95% CI) | Number of Studies (Participants) | Downgrading Factors | GRADE Certainty |
| --- | --- | --- | --- | --- | --- |
| Objective Response Rate (ORR) | 18% (illustrative Risk) | RR = 0.82 (0.57–1.17) | 5 studies (489 participants) | -1 for imprecision (small number of studies; 95% CI crosses 1.0) | Moderate |
| Disease Control Rate (DCR) | 70% (illustrative Risk) | RR = 1.01 (0.67–1.53) | 2 studies (198 participants) | -1 for imprecision (only 2 studies; small sample);-1 for inconsistency (potential publication bias per Begg’s test, p=0) | Low |
| Progression-Free Survival (PFS) | Median 4.5 months | HR = 0.79 (0.45–1.39) | 5 studies (512 participants) | -1 for imprecision (small number of studies; 95% CI crosses 1.0);-1 for inconsistency (I²=79%, high heterogeneity; sensitivity analysis shows Takahashi 2023 drives variability) | Low |
| Overall Survival (OS) | Median 10 months | HR = 0.81 (0.42–1.55) | 5 studies (512 participants) | -1 for imprecision (small number of studies; 95% CI crosses 1.0);-1 for inconsistency (I²=87%, high heterogeneity; sensitivity analysis shows Takahashi 2023 drives variability) | Low |
| Adverse Events (AEs) | 90% (illustrative High Burden) | RR = 1.03 (0.92–1.16) | 10 studies (810 participants) | No downgrades (low RoB; I²=26% no significant inconsistency; large sample/study number; no publication bias) | High |
