# Supplementary material for: The efficacy and safety of ATR inhibitors in the treatment of solid tumors: a systematic review and meta-analysis
Source: Front Oncol. 2025 Dec 2;15:1706837. doi: 10.3389/fonc.2025.1706837 (PMC12705417; doi:10.3389/fonc.2025.1706837)
Supplement: Supplementary file 2 [file DataSheet2.docx]

search strategy

PubMed:

(((((((((((((((((((((ATR inhibitor[Title/Abstract]) OR (ATR kinase inhibitor[Title/Abstract])) OR (Ataxia Telangiectasia and Rad3-Related Protein[Title/Abstract])) OR (Ceralasertib[Title/Abstract])) OR (AZD6738[Title/Abstract])) OR (Berzosertib[Title/Abstract])) OR (M6620[Title/Abstract])) OR (Elimusertib[Title/Abstract])) OR (BAY1895344[Title/Abstract])) OR (gartisertib[Title/Abstract])) OR (VX-803[Title/Abstract])) OR (Camonsertib[Title/Abstract])) OR (RP-3500[Title/Abstract])) OR (IMP9064[Title/Abstract])) OR (ATRN-119[Title/Abstract])) OR (AZ20[Title/Abstract])) OR (VE-821[Title/Abstract])) OR (Schisandrin B[Title/Abstract])) OR (NU6027[Title/Abstract])) OR (M1774[Title/Abstract])) OR (ART0380[Title/Abstract])) AND (((((((((((((((((((Neoplasms[MeSH Terms])) OR (Tumors[Title/Abstract])) OR (Neoplasias[Title/Abstract])) OR (Neoplasm[Title/Abstract])) OR (Tumor[Title/Abstract])) OR (Cancer[Title/Abstract])) OR (Cancers[Title/Abstract])) OR (Malignant Neoplasm[Title/Abstract])) OR (Malignancy[Title/Abstract])) OR (Malignancies[Title/Abstract])) OR (Malignant Neoplasms[Title/Abstract])) OR (Neoplasm, Malignant[Title/Abstract])) OR (Neoplasms, Malignant[Title/Abstract])) OR (Benign Neoplasms[Title/Abstract])) OR (Neoplasms, Benign[Title/Abstract])) OR (Neoplasm, Benign[Title/Abstract])) OR (Benign Neoplasm[Title/Abstract])) OR (Neoplasia[Title/Abstract]))

Cochrane Library：

((ATR inhibitor):ti,ab,kw OR (ATR kinase inhibitor):ti,ab,kw OR (Ataxia Telangiectasia and Rad3-Related Protein):ti,ab,kw OR (Ceralasertib):ti,ab,kw OR (AZD6738):ti,ab,kw OR (Berzosertib):ti,ab,kw OR (M6620):ti,ab,kw OR (Elimusertib):ti,ab,kw OR (BAY1895344):ti,ab,kw OR (gartisertib):ti,ab,kw OR (VX-803):ti,ab,kw OR (Camonsertib):ti,ab,kw OR (RP-3500):ti,ab,kw OR (IMP9064):ti,ab,kw OR (ATRN-119):ti,ab,kw OR (AZ20):ti,ab,kw OR (VE-821):ti,ab,kw OR (Schisandrin B):ti,ab,kw OR (NU6027):ti,ab,kw OR (M1774):ti,ab,kw OR (ART0380):ti,ab,kw) AND ((Neoplasms):ti,ab,kw OR (Benign Neoplasms):ti,ab,kw OR (Neoplasms, Benign):ti,ab,kw OR (Benign Neoplasm):ti,ab,kw OR (Neoplasm, Benign):ti,ab,kw OR (Tumors):ti,ab,kw OR (Neoplasias):ti,ab,kw OR (Neoplasia):ti,ab,kw OR (Neoplasm):ti,ab,kw OR (Tumor):ti,ab,kw OR (Malignancy):ti,ab,kw OR (Cancers):ti,ab,kw OR (Neoplasms, Malignant):ti,ab,kw OR (Neoplasm, Malignant):ti,ab,kw OR (Malignant Neoplasms):ti,ab,kw OR (Malignancies):ti,ab,kw OR (Malignant Neoplasm):ti,ab,kw OR (Cancer):ti,ab,kw)

Web of Science:

(TS=(ATR inhibitor OR ATR kinase inhibitor OR “Ataxia Telangiectasia and Rad3-Related Protein” OR Ceralasertib OR AZD6738 OR Berzosertib OR M6620 OR Elimusertib OR BAY1895344 OR Gartisertib OR VX-803 OR Camonsertib OR RP-3500 OR IMP9064 OR ATRN-119 OR AZ20 OR VE-821 OR Schisandrin B OR NU6027 OR M1774 OR ART0380)) AND (TS=(Neoplasms OR Benign Neoplasms OR Neoplasms, Benign OR Benign Neoplasm OR Neoplasm, Benign OR Tumors OR Neoplasias OR Neoplasia OR Neoplasm OR Tumor OR Malignancy OR Cancers OR Neoplasms, Malignant OR Neoplasm, Malignant OR Malignant Neoplasms OR Malignancies OR Malignant Neoplasm OR Cancer))

Embase:

(‘ATR inhibitor’:ab,ti OR ‘ATR kinase inhibitor’:ab,ti OR ‘Ataxia Telangiectasia and Rad3-Related Protein’:ab,ti OR ‘Ceralasertib’:ab,ti OR ‘AZD6738’:ab,ti OR ‘Berzosertib’:ab,ti OR ‘M6620’:ab,ti OR ‘Elimusertib’:ab,ti OR ‘BAY1895344’:ab,ti OR ‘Gartisertib’:ab,ti OR ‘VX-803’:ab,ti OR ‘Camonsertib’:ab,ti OR ‘RP-3500’:ab,ti OR ‘IMP9064’:ab,ti OR ‘ATRN-119’:ab,ti OR ‘AZ20’:ab,ti OR ‘VE-821’:ab,ti OR ‘Schisandrin B’:ab,ti OR ‘NU6027’:ab,ti OR ‘M1774’:ab,ti OR ‘ART0380’:ab,ti) AND (‘Neoplasms’:ab,ti OR ‘Benign Neoplasms’:ab,ti OR ‘Neoplasms, Benign’:ab,ti OR ‘Benign Neoplasm’:ab,ti OR ‘Neoplasm, Benign’:ab,ti OR ‘Tumors’:ab,ti OR ‘Neoplasias’:ab,ti OR ‘Neoplasia’:ab,ti OR ‘Neoplasm’:ab,ti OR ‘Tumor’:ab,ti OR ‘Malignancy’:ab,ti OR ‘Cancers’:ab,ti OR ‘Neoplasms, Malignant’:ab,ti OR ‘Neoplasm, Malignant’:ab,ti OR ‘Malignant Neoplasms’:ab,ti OR ‘Malignancies’:ab,ti OR ‘Malignant Neoplasm’:ab,ti OR ‘Cancer’:ab,ti)

CNKI (中国知网):

（主题：’肿瘤’ + ‘癌' + '瘤'）AND（主题：'ATR inhibitor' + 'ATR kinase inhibitor' + 'Ataxia Telangiectasia and Rad3-Related Protein' + 'Ceralasertib' + 'AZD6738' + 'Berzosertib' + 'M6620' + 'Elimusertib' + 'BAY1895344' + 'Gartisertib' + 'VX-803' + 'Camonsertib' + 'RP-3500' + 'IMP9064' + 'ATRN-119' + 'AZ20' + 'VE-821' + 'Schisandrin B' + 'NU6027' + 'M1774' + 'ART0380'）

Wanfang Data (万方数据):

主题: (ATR inhibitor OR ATR kinase inhibitor OR Ataxia Telangiectasia and Rad3-Related Protein OR Ceralasertib OR AZD6738 OR Berzosertib OR M6620 OR Elimusertib OR BAY1895344 OR Gartisertib OR VX-803 OR Camonsertib OR RP-3500 OR IMP9064 OR ATRN-119 OR AZ20 OR VE-821 OR Schisandrin B OR NU6027 OR M1774 OR ART0380) and 主题: (肿瘤 OR 癌 OR 瘤)

VIP Database (维普网):

((K=("ATR inhibitor" OR "ATR kinase inhibitor" OR "Ataxia Telangiectasia and Rad3-Related Protein" OR "Ceralasertib" OR "AZD6738" OR "Berzosertib" OR "M6620" OR "Elimusertib" OR "BAY1895344" OR "Gartisertib" OR "VX-803" OR "Camonsertib" OR "RP-3500" OR "IMP9064" OR "ATRN-119" OR "AZ20" OR "VE-821" OR "Schisandrin B" OR "NU6027" OR "M1774" OR "ART0380") OR T=("ATR inhibitor" OR "ATR kinase inhibitor" OR "Ataxia Telangiectasia and Rad3-Related Protein" OR "Ceralasertib" OR "AZD6738" OR "Berzosertib" OR "M6620" OR "Elimusertib" OR "BAY1895344" OR "Gartisertib" OR "VX-803" OR "Camonsertib" OR "RP-3500" OR "IMP9064" OR "ATRN-119" OR "AZ20" OR "VE-821" OR "Schisandrin B" OR "NU6027" OR "M1774" OR "ART0380") OR R=("ATR inhibitor" OR "ATR kinase inhibitor" OR "Ataxia Telangiectasia and Rad3-Related Protein" OR "Ceralasertib" OR "AZD6738" OR "Berzosertib" OR "M6620" OR "Elimusertib" OR "BAY1895344" OR "Gartisertib" OR "VX-803" OR "Camonsertib" OR "RP-3500" OR "IMP9064" OR "ATRN-119" OR "AZ20" OR "VE-821" OR "Schisandrin B" OR "NU6027" OR "M1774" OR "ART0380"))) AND (K=("肿瘤" OR "癌" OR "瘤") OR T=("肿瘤" OR "癌" OR "瘤") OR R=("肿瘤" OR "癌" OR "瘤"))

Sinomed (中国生物医学文献数据库):

(“肿瘤”[常用字段:智能] OR “癌”[常用字段:智能] OR “瘤”[常用字段:智能]) AND (“ATR inhibitor”[常用字段：智能] OR “ATR kinase inhibitor”[常用字段：智能] OR “Ataxia Telangiectasia and Rad3-Related Protein”[常用字段：智能] OR “Ceralasertib”[常用字段：智能] OR “AZD6738”[常用字段：智能] OR “Berzosertib”[常用字段：智能] OR “M6620”[常用字段：智能] OR “Elimusertib”[常用字段：智能] OR “BAY1895344”[常用字段：智能] OR “Gartisertib”[常用字段：智能] OR “VX-803”[常用字段：智能] OR “Camonsertib”[常用字段：智能] OR “RP-3500”[常用字段：智能] OR “IMP9064”[常用字段：智能] OR “ATRN-119”[常用字段：智能] OR “AZ20”[常用字段：智能] OR “VE-821”[常用字段：智能] OR “Schisandrin B”[常用字段：智能] OR “NU6027”[常用字段：智能] OR “M1774”[常用字段：智能] OR “ART0380”[常用字段：智能])

The search strategy for meeting abstracts (ASCO, AACR, ESMO) is: (ATR inhibitor OR Ceraresterib OR AZD6738 OR Berzosertib OR M6620 OR Elimusertib OR Camonsertib OR RP-3500) AND (tumor OR cancer OR tumor). The last search date for conference abstracts was September, 2025, and the time range for abstracts was 2020–2025 (to avoid outdated data and minimize overlap with published studies).
